# Y_6_, an Epigallocatechin Gallate Derivative, Reverses ABCG2-Mediated Mitoxantrone Resistance

**DOI:** 10.3389/fphar.2018.01545

**Published:** 2019-01-10

**Authors:** Rui-Qiang Zhao, Yan Wen, Pranav Gupta, Zi-Ning Lei, Chao-Yun Cai, Gang Liang, Dong-Hua Yang, Zhe-Sheng Chen, Yu-An Xie

**Affiliations:** ^1^The Affiliated Tumor Hospital of Guangxi Medical University, Nanning, China; ^2^Department of Biochemistry and Molecular Biology, School of Preclinical Medicine, Guangxi Medical University, Nanning, China; ^3^Department of Pharmaceutical Sciences, College of Pharmacy and Health Sciences, St. John’s University, Queens, NY, United States; ^4^Department of Pharmacy, The First Affiliated Hospital of Guangxi Medical University, Nanning, China; ^5^College of Pharmacy, Guangxi Medical University, Nanning, China

**Keywords:** epigallocatechin gallate (EGCG), 5, 3′, 4′, 3^′′^, 4^′′^, 5^′′^-6-*O*-ethyl-EGCG (Y_6_), ABC transporter, ABCG2, multidrug resistance

## Abstract

Multidrug resistance is reported to be related to the transmembrane transportation of chemotherapeutic drugs by adenosine triphosphate-binding cassette (ABC) transporters. ABC subfamily G member 2 (ABCG2) is a member of the ABC transporter superfamily proteins, which have been implicated as a key contributor to the development of multidrug resistance in cancers. A new epigallocatechin gallate derivative, Y_6_ was synthesized in our group. Our previous study revealed that Y_6_ increased the sensitivity of drug-resistant cells to doxorubicin, which was associated with down-regulation of P-glycoprotein expression. In this study, we further determine whether Y_6_ could reverse ABCG2-mediated multidrug resistance. Results showed that, at non-toxic concentrations, Y_6_ significantly sensitized drug-selected non-small cell lung cancer cell line NCI-H460/MX20 to substrate anticancer drugs mitoxantrone, SN-38, and topotecan, and also sensitized ABCG2-transfected cell line HEK293/ABCG2-482-R2 to mitoxantrone and SN-38. Further study demonstrated that Y_6_ significantly increased the accumulation of [^3^H]-mitoxantrone in NCI-H460/MX20 cells by inhibiting the transport activity of ABCG2, without altering the expression levels and the subcellular localization of ABCG2. Furthermore, Y_6_ stimulated the adenosine triphosphatase activity with a concentration-dependent pattern under 20 μM in membranes overexpressing ABCG2. In addition, Y_6_ exhibited a strong interaction with the human ABCG2 transporter protein. Our findings indicate that Y_6_ may potentially be a novel reversal agent in ABCG2-positive drug-resistant cancers.

## Introduction

Multidrug resistance (MDR) to chemotherapeutic drugs could be found in various types of cancer cells (Fletcher et al., 2010). A wide range of structurally different chemotherapeutic drugs have been shown to be substrates of ATP-binding cassette (ABC) transporter proteins. Powered by the energy from ATP hydrolysis, ABC-transporters have become a significant impediment in cancer therapy. In total, 48 different ABC transporters were identified in the human genome, divided to seven subfamilies (ABCA-ABCG) due to structural similarities. Among all subfamilies, P-glycoprotein (P-gp/ABCB1) and breast cancer resistance protein (BCRP/ABCG2) are major transporters in MDR (Sodani et al., 2012).

ABCG2 is also known as mitoxantrone resistance-associated protein (MXR) or placenta-specific ATP-binding cassette transporter (ABCP) (Miyake et al., 1999; Dean and Allikmets, 2001). The ABCG2 protein has a molecular weight of 72-kDa. It is a half transporter with one nucleotide binding domain (NBD) and one transmembrane domain (TMD) and functions in the form of homodimer or an oligomer (Taylor et al., 2017). ABCG2 transporter is specifically distributed in the plasma membrane. Normally, it is highly expressed in the colon epithelium, the apical surface of small intestines, the canalicular membrane of liver and bile duct, cortical tubules of the kidney and prostate epithelium, and at the luminal surfaces of microvessel endothelium of human brain (Maliepaard et al., 2001; Fetsch et al., 2006). Such distribution results in alteration of the absorption, distribution, metabolism, and elimination of drugs since ABCG2 performs compound transmembrane transport on secretory surfaces of organs. ABCG2 could transport a variety of anti-neoplastic drugs such as mitoxantrone, topotecan, irinotecan, doxorubicin, daunorubicin, 9-aminocamptothecin, and epirubicin as its substrates (Doyle and Ross, 2003).

A mutation at position 482 (Arg or R) of ABCG2 produces a distinct substrate preference (Chen et al., 2003), which results in different drug-binding and drug-efflux capacity of the transporter (Honjo et al., 2001; Pozza et al., 2006; Dai et al., 2009). For example, after Arg at position 482 was replaced by threonine (Thr or T) or glycine (Gly or G), the substrate specificity was changed in both mutant and wild-type variants. Mitoxantrone and major nucleoside inhibitors are common substrates of wild-type and mutant ABCG2, while daunorubicin is the substrate of mutant Gly and Thr variants and is not a substrate of the wild-type ABCG2 (Ejendal and Hrycyna, 2002; Mao and Unadkat, 2005; Ejendal et al., 2006).

Multidrug resistance in acute myeloid leukemia (AML), non-small cell lung cancer (NSCLC), colon carcinoma and breast cancer were also reported to be strongly correlated with the overexpression of ABCG2 (Steinbach et al., 2002; van den Heuvel-Eibrink et al., 2002; Nakanishi and Ross, 2012). Notably, ABCG2 is overexpressed only in subpopulations of AML specimens (Abbott et al., 2002; Suvannasankha et al., 2004). The overexpression of ABCG2 in these subpopulations of stem cells was also reported among other tumors such as neuroblastomas, Ewing sarcomas, breast cancer, small cell lung cancer, and glioblastomas (Hirschmann-Jax et al., 2004). These stem cells with overexpression of ABCG2 may play an important role in resistance to chemotherapeutic drugs.

**FIGURE 1:**
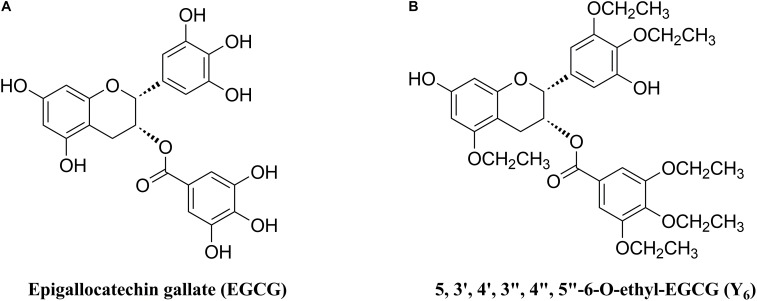
Chemical structures of **(A)** EGCG and **(B)** Y_6_.

As one of the MDR reversal modulators, epigallocatechin gallate (EGCG) was revealed to down-regulate P-gp and BCRP in a tamoxifen resistant cancer cell line (Farabegoli et al., 2010). Furthermore, EGCG (Figure 1A) was reported to significantly inhibit proliferation of a drug-resistant cancer cell line, BEL-7404/DOX, *in vitro* when co-administered with doxorubicin (Liang et al., 2010). However, the application of EGCG was limited due to an unstable chemical profile that could be subjected to rapid oxidation and short duration of action because of multiple phenolic hydroxyl groups (Lee et al., 2002). Inspired by the structure of EGCG, we synthesized Y_6_ (Figure 1B), an ethylation product of EGCG. Y_6_ has been evaluated as a reversal agent that specifically reverses ABC transporter-mediated MDR *in vitro* (Wen et al., 2017). In this study, we determined the potential effect of Y_6_ as a reversal agent that re-sensitizes ABCG2-mediated MDR *in vitro*.

## Materials and Methods

### Reagents

Y_6_ (purity 96.87%) was synthesized in our research group and was made in a 10 mM stock solution in dimethylsulfoxide (DMSO). Dulbecco’s modified Eagle’s medium (DMEM), penicillin/streptomycin, trypsin 0.25%, fetal bovine serum (FBS) and bovine calf serum were purchased from Hyclone (GE Healthcare Life Science, Pittsburgh, PA, United States). Mitoxantrone, SN-38, topotecan, and cisplatin were products from Sigma-Aldrich (St. Louis, MO, United States). Fumitremorgin C (FTC) was provided by Dr. Susan E. Bates from NIH (Bethesda, MD, United States). [^3^H]-Mitoxantrone was purchased from Moravek Biochemicals, Inc. (Brea, CA, United States). Mouse monoclonal ABCG2 antibody and mouse monoclonal β-actin antibody were purchased from Gene Tex, Inc. (Irvine, CA, United States). Goat anti-mouse IgG secondary antibody conjugated with Alexa Fluor 488 and 4′,6-diamidino-2-phenylindole (DAPI) were purchased from Thermo Fisher Scientific, Inc. (Rockford, IL, United States). The ATPase assay kit and membrane vesicles were purchased from BD Biosciences (San Jose, CA, United States). PBS and trypsin were purchased from Bosterbio (Pleasanton, CA, United States). RIPA lysis buffer, protein loading buffer, BCA protein assay kit, SDS-PAGE gel preparation kit, protein marker, western transfer buffer, SDS-PAGE running buffer, primary/secondary antibody dilution buffer were purchased from Sigma-Aldrich (St. Louis, MO, United States).

### Cell Lines and Cell Culture

Non-small cell lung cancer cell line NCI-H460 and its mitoxantrone-selected ABCG2-overexpressing NCI-H460/MX20 cells were used in the ABCG2 reversal study. NCI-H460/MX20 cells were cultured in a medium containing 20 nM of mitoxantrone. HEK293/pcDNA3.1 and wild-type HEK293/ABCG2-482-R2 were established by transfecting HEK293 cells with either an empty pcDNA3.1 vector or a vector containing full length ABCG2 with Arg at position 482. HEK293/ABCG2-482-R2 cells were cultured in a medium containing 2 mg/mL of G418. All the cell lines were maintained in DMEM containing 10% (v/v) FBS and 1% (v/v) penicillin/streptomycin in a constant temperature incubator containing 5% CO_2_ at 37°C.

### Cytotoxicity by MTT Assay

The viability of NCI-H460 and NCI-H460/MX20 cells or HEK293/pcDNA3.1 and HEK293/ABCG2-482-R2 cells to chemotherapeutic drugs was measured for the ABCG2 reversal study using the MTT assay. Cells (5000 cells per well) were seeded into 96-well microplates with 160 μL per well and cultured overnight. Mitoxantrone, SN-38, topotecan, and cisplatin were diluted with PBS to a series of various concentrations and added to designated wells (20 μL per well) with or without the reversal agents (Y_6_ and FTC) (20 μL per well). Cells were incubated for 72 h at 37°C. Then, 20 μl of MTT solution (4 mg/mL, in PBS) was added into each well and incubated for 4 h at 37°C in dark. Then the supernatant medium was discarded and 100 μl of DMSO was added to each well for formazan dissolution. Finally, the light absorbance at 490 nm was measured using the OPSYS microplate reader (Dynex Technology, Chantilly, VA, United States). FTC was used as positive reversal agent of ABCG2.

### Western Blotting Analysis

NCI-H460/MX20 cells were treated with or without Y_6_ for 72 h with different drug concentrations (5, 10, 15 μM). In addition, NCI-H460/MX20 cells were also treated with or without 10 μM of Y_6_ for different time periods (24, 48, 72 h). Then cells were collected and lysed in ice-cold lysis buffer containing 50 mM Tris (pH 7.4), 150 mM NaCl, 1% Triton X-100, 0.1% SDS, 1 mM EDTA, and 1× phosphatase inhibitor cocktail on ice for 20 min. Cell lysates were centrifuged at 4°C at 13,000 rpm/min for 10 min. Subsequently, the supernatant was collected in Eppendorf tubes and protein concentrations were determined by bicinchoninic acid (BCATM)-based protein assay (Thermo Scientific, Rockford, IL, United States). Equal amount of protein was loaded and separated by SDS-polyacrylamide gel electrophoresis and transferred to a polyvinylidene fluoride (PVDF) membrane through electrophoresis. The PVDF membrane was submerged in 5% skim milk for 1 h, and then was incubated with primary monoclonal antibodies (ABCG2 at 1:500 dilution or β-actin at 1:1000 dilution) overnight at 4°C. After the membrane was washed with the TBST buffer, the membrane was incubated with HRP (horseradish peroxidase)-conjugated secondary antibody (1:1000 dilution) at room temperature for 2 h. The washing with TBST buffer, the membrane was exposed to Infrared Imaging System to visualize the bands.

### Immunofluorescence of ABCG2

Equal amounts of NCI-H460 and NCI-H460/MX20 cells were seeded in sterile coated 24-well plates and were cultured overnight. NCI-H460/MX20 cells were treated with or without 10 μM of Y_6_ for different time periods (24, 48, 72 h). Then the cells were fixed in 4% paraformaldehyde for 15 min, permeabilized by 0.25% Triton X-100 for 15 min and then blocked with 6% BSA for 1 h. Cells were further incubated with mouse monoclonal antibody against ABCG2 (1:200) overnight, and then were submerged in Alexa Fluor 488 conjugated secondary antibody (1:2000) solution in the dark for 2 h to localize ABCG2. 2-(4-amidinophenyl)-6-indolecarbamidene dihydrochloride (DAPI) solution was used to counterstain the nuclei in dark. Images were taken with an inverted IX70 microscope (Olympus, Center Valley, PA, United States) following our previous protocol (Guo et al., 2014).

### [^3^H]-Mitoxantrone Accumulation Assay

The accumulation of [^3^H]-mitoxantrone in NCI-H460 and NCI-H460/MX20 was measured in the presence or absence of inhibitors (FTC or Y_6_). The cells (5 × 10^6^) in each centrifuge tube were trypsinized, resuspended, and pre-incubated with PBS, Y_6_ (5 and 10 μM), or FTC (5 μM) for 2 h at 37°C. After the cell suspension was centrifuged at 1500 rpm/min for 5 min, the supernatant was discarded. Subsequently, cells were resuspended in medium containing 0.1 μM [^3^H]-mitoxantrone at 37°C for 2 h in the presence or absence of inhibitors following above treatment. Cells were washed three times with ice-cold PBS. Then, cells were placed in 5 ml scintillation liquid and their radioactivity was measured in the Packard TRI-CARB 1900CA liquid scintillation analyzer (Packard Instrument, Downers Grove, IL, United States) (Sun et al., 2012; Patel et al., 2013). FTC was used as the positive reversal agent of ABCG2.

### [^3^H]-Mitoxantrone Efflux Assay

Mitoxantrone drug efflux in NCI-H460 or NCI-H460/MX20 was measured. The cells were pretreated with PBS, Y_6_ (5 and 10 μM) or FTC (5 μM) for 2 h at 37°C. Then radioactive substrate [^3^H]-mitoxantrone was added and the cells were further incubated for 2 h. Cell suspension was centrifuged at 1500 rpm/min for 5 min and the supernatant was discarded. The cells were re-supplemented with fresh medium with or without a reversal agent. After 0, 30, 60, and 120 min, the aliquots of cells were removed and washed three times with ice-cold PBS immediately. Radioactivity was then measured as described in the accumulation assay above.

### ABCG2 ATPase Assay

The vanadate-sensitive ATPase activity of ABCG2 was performed by an ATPase assay kit from BD Biosciences (San Jose, CA, United States) as previously described (Zhang et al., 2016). Briefly, the ABCG2 membrane vesicles were incubated in ATPase buffer with or without vanadate for 5 min at 37°C. Y_6_ was then added to the assay buffer in concentration gradient (0–40 μM). The mixtures were incubated for 5 min at 37°C. Then Mg-ATP solution was added to assay buffer and was incubated for 20 min at 37°C. The mixture was added with 100 μL of 5% SDS solution and then its light absorption was detected at 880 nm using a spectrophotometer.

### Molecular Docking of Y_6_ With ABCG2 Model

Molecular modeling was performed with Maestro v11.1 (Schroñdinger, LLC, New York, NY, United States 2017) software (Zhang et al., 2017). The protein preparation of wild-type human ABCG2 (PDB ID: 6FFC) (Jackson et al., 2018) was essentially performed and the grid (30 Å) was generated by selecting the same binding pocket as the two substrates (MZ29) in TMD. After that, the structure of Y_6_ was built and prepared. Compound Y_6_ with best-scored conformation was obtained through Glide XP docking then was used to generate receptor grid for induced-fit docking (IFD). The IFD protocol with default parameters was performed and the docking score (kcal/mol) was obtained.

### Statistical Analysis

The data were analyzed using a *t*-test method. All values represent the mean ± standard deviation SD of three independent experiments performed in triplicate. The priori *p*-value for significance was *p* < 0.05.

## Results

### Y_6_ Sensitized ABCG2-Overexpressing Cells to Chemotherapeutic Drugs

**FIGURE 2:**
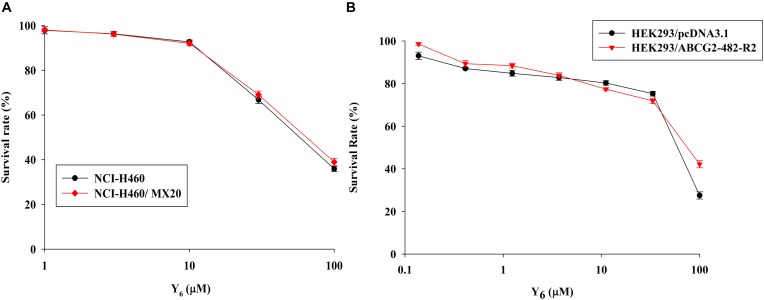
The cytotoxicity of Y_6_ in parental and ABCG2-overexpressing cells. **(A)** Cytotoxicity of Y_6_ was evaluated in parental NCI-H460 and ABCG2-overexpressing NCI-H460/MX20 cells. **(B)** Cytotoxicity of Y_6_ was evaluated in transfected HEK293/pcDNA3.1 and ABCG2-overexpressing HEK293/ABCG2-482-R2 cells. Cells were incubated with different concentration of Y_6_ for 72 h. Survival rate was determined by MTT assay. Representative curves were shown as cell survival rate verses concentration of compounds. Points and error bars display the mean and SD.

In order to investigate the reversal effects of Y_6_ on drug-resistant cells, we first examined the sensitivity of ABCG2-overexpressing cells to Y_6_. Based on the results from the cytotoxicity assay, two non-toxic concentrations of Y_6_ (5.0 and 10.0 μM) were selected for further experimentation (Figures 2A,B).

In the sensitization experiment, mitoxantrone-selected NCI-H460/MX20 cells showed a much higher IC_50_ value to ABCG2 substrates (mitoxantrone, SN-38, and topotecan) than that in parental NCI-H460 cells. Y_6_ at both 5.0 and 10.0 μM were able to significantly increase the sensitivity of NCI-H460/MX20 cells to mitoxantrone, SN-38, and topotecan. A significant reduction in IC_50_ values was observed with the treatment of Y_6_ in NCI-H460/MX20 cells as shown in Table 1. Meanwhile, no significant changes in IC_50_ values were observed in parental NCI-H460 cells. Similarly, Y_6_ also increased the sensitivity of transfected HEK293/ABCG2-482-R2 cells, which had a much higher IC_50_ value to ABCG2 substrates than that in parental HEK293/pcDNA3.1 cells. Significant decrease in IC_50_ values of mitoxantrone and SN-38 was observed in Y_6_-present treatment of transfected HEK293/ABCG2-482-R2 cells as compared to Y_6_-absent treatment, and no significant change was observed in HEK293/pcDNA3.1 cells as shown in Table 2. Uniformly, the efficacy of Y_6_ showed a concentration-dependent pattern. Cisplatin, which is not a substrate of ABCG2, was used as a negative control. FTC at 5.0 μM was used as a positive control to evaluate the effects of Y_6_.

**Table 1:** Reversal effects of Y_6_ to NCI-H460 and NCI-H460/MX20 cell lines.

Compounds	IC_50_± SD^a^ (nM)			
	NCI-H460	(RF^b^)	NCI-H460/MX20	(RF)
Mitoxantrone	45.11 ± 1.79	1.0	2670.33 ± 105.10	59.2
+ Y_6_ 5.0 μM	34.99 ± 4.33	0.8	1268.67 ± 87.85	28.1^∗^
+ Y_6_ 10.0 μM	35.69 ± 5.67	0.8	414.16 ± 6.86	9.2^∗^
+ FTC 5.0 μM	27.51 ± 2.67	0.6	532.04 ± 19.04	11.8^∗^
				
SN-38	446.21 ± 41.04	1.0	10614.67 ± 775.84	23.8
+ Y_6_ 5.0 μM	377.84 ± 22.25	0.8	1831.67 ± 116.62	4.1^∗^
+ Y_6_ 10.0 μM	310.97 ± 30.29	0.7	944.61 ± 28.50	2.1^∗^
+ FTC 5.0 μM	221.18 ± 17.71	0.5	1048.89 ± 57.92	2.4^∗^
				
Topotecan	174.39 ± 8.92	1.0	9041.33 ± 535.04	51.8
+ Y_6_ 5.0 μM	60.85 ± 4.55	0.3	1205.67 ± 40.02	6.9^∗^
+ Y_6_ 10.0 μM	57.78 ± 4.83	0.3	406.49 ± 5.97	2.3^∗^
+ FTC 5.0 μM	61.98 ± 5.18	0.4	629.64 ± 26.71	3.6^∗^

**Compounds**	**IC_50_± SD (μM)**			
	**NCI-H460**	**(RF)**	**NCI-H460/MX20**	**(RF)**

Cisplatin	1.98 ± 0.07	1.0	1.77 ± 0.15	0.9
+ Y_6_ 5.0 μM	1.60 ± 0.07	0.8	1.88 ± 0.13	0.9
+ Y_6_ 10.0 μM	1.85 ± 0.16	0.9	1.85 ± 0.13	0.9
+ FTC 5.0 μM	1.85 ± 0.03	0.9	1.77 ± 0.03	0.9

*^a^IC_50_ values are represented as mean ± SD of three independent experiments performed in triplicate; ^b^Values represent the resistance fold (RF) calculated by dividing IC_50_ values of anticancer drug in NCI-H460 and NCI-H460/MX20 cells in presence or absence of reversal agent by the IC_50_ value of NCI-H460 cells without reversal agent. ^∗^p < 0.05 versus no reversal agent group.*

**Table 2:** Reversal effects of Y_6_ to HEK293/pcDNA3.1 and HEK293/ ABCG2-482-R2 cell lines.

Compounds	IC_50_± SD^a^ (nM)			
	HEK293/ pcDNA3.1	(RF^b^)	HEK293/ ABCG2-482-R2	(RF)
Mitoxantrone	76.06 ± 4.14	1.0	662.90 ± 45.40	8.7
+ Y_6_ 5.0 μM	67.91 ± 5.44	0.9	184.22 ± 11.47	2.4^∗^
+ Y_6_ 10.0 μM	41.05 ± 4.24	0.5	84.31 ± 3.02	1.1^∗^
+ FTC 5.0 μM	67.39 ± 7.37	0.9	94.77 ± 3.87	1.2^∗^
				
SN-38	61.92 ± 1.41	1.0	571.30 ± 31.80	9.2
+ Y_6_ 5.0 μM	62.32 ± 2.59	1.0	152.24 ± 13.88	2.5^∗^
+ Y_6_ 10.0 μM	56.64 ± 7.25	0.9	79.23 ± 7.14	1.3^∗^
+ FTC 5.0 μM	57.41 ± 4.11	0.9	80.09 ± 7.07	1.3^∗^

**Compounds**	**IC_50_± SD (μM)**			
	**HEK293/pcDNA3.1**	**(RF)**	**HEK293/ ABCG2-482-R2**	**(RF)**

Cisplatin	1.80 ± 0.03	1.0	1.57 ± 0.10	0.9
+ Y_6_ 5.0 μM	1.81 ± 0.02	1.0	1.75 ± 0.01	1.0
+ Y_6_ 10.0 μM	1.80 ± 0.11	1.0	1.53 ± 0.12	0.9
+ FTC 5.0 μM	1.84 ± 0.05	1.0	1.69 ± 0.10	0.9

^a^IC_50_ values are represented as mean ± SD of three independent experiments performed in triplicate; ^b^RF calculated by dividing IC_50_ values of anticancer drug in HEK293/pcDNA3.1 and HEK293/ ABCG2-482-R2 cells in presence or absence of reversal agent by the IC_50_ value of HEK293/pcDNA3.1 cells without reversal agent. ^∗^p < 0.05 versus no reversal agent group.

Based on the above results, it appeared that Y_6_ could significantly reverse ABCG2-mediated MDR in both NCI-H460/MX20 and HEK293/ABCG2-482-R2 cells.

### Y_6_ Had No Effect on the Protein Expression of ABCG2 in NCI-H460/MX20 Cells

**FIGURE 3:**
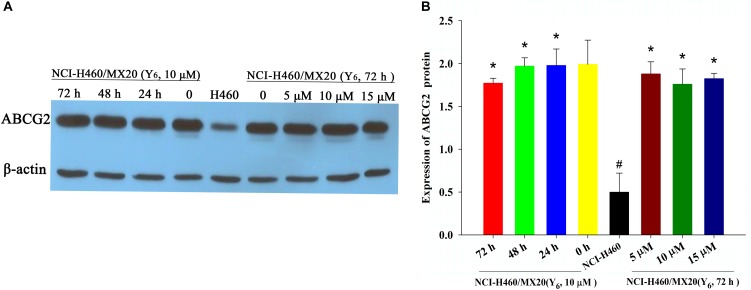
**(A)** The expression of ABCG2 in NCI-H460 and NCI-H460/MX20 cells with different treatments. Effect of Y_6_ at 10 μM on expression levels of ABCG2 in NCI-H460 and NCI-H460/MX20 cells at 0, 24, 48, and 72 h; or 0, 5, 10, 15 μM of Y_6_ after incubation for 72 h. **(B)** Quantified ABCG2 expression in NCI-H460 and NCI-H460/MX20 cells with different treatments. Values represent means ± SDs of at least three independent experiments performed in triplicate; ^∗^*p* > 0.05 vs. the NCI-H460/MX20 cells without treatment on the ABCG2 protein; ^#^*p* < 0.05 vs. the NCI-H460/MX20 cells without treatment on the ABCG2 protein.

To determine the reversal mechanism of Y_6_ on ABCG2-mediated MDR in cancer cells, Western blot analysis was performed using the parental NCI-H460 cells and the drug-selective NCI-H460/MX20 cells. NCI-H460/MX20 cells were incubated with either different concentrations of Y_6_ (0, 5, 10, 15 μM for 72 h) or different incubation time (0, 24, 48, 72 h at 10 μM). No significant changes were observed in the expression level of ABCG2 protein in NCI-H460/MX20 cells treated with Y_6_ compared to Y_6_-absent treatment group (Figures 3A,B). The results indicated that the MDR reversal mechanism of Y_6_ was not relative to the expression level of ABCG2.

### Y_6_ Did Not Alter the Subcellular Localization of ABCG2 in NCI-H460/MX20 Cells

**FIGURE 4:**
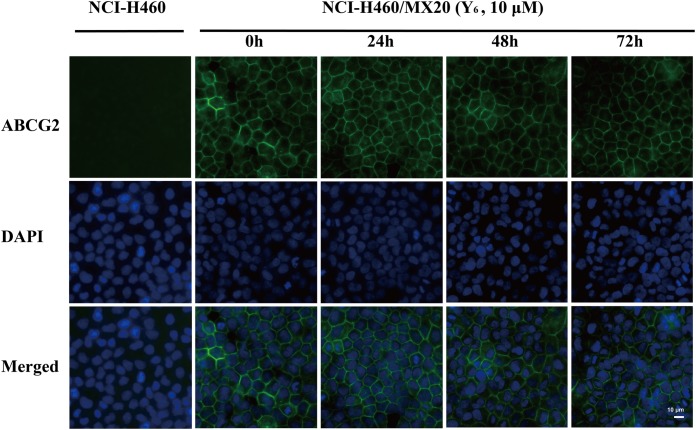
Effect of Y_6_ at 10 μM and different times on the subcellular localization of ABCG2. Scale bar: 10 μM, DAPI (blue) counterstains the nuclei. ABCG2 staining is shown in green.

As a transmembrane protein, ABCG2 could also be affected by protein localization. Thus, effect of Y_6_ on ABCG2 protein cellular localization was determined with immunofluorescence assay. As is shown in Figure 4, Y_6_ did not trigger the internalization of ABCG2 in NCI-H460/MX20 cells after incubating with 10 μM of Y_6_ for 0, 24, 48, and 72 h. The results indicated that the MDR reversal mechanism of Y_6_ was not induced by altering the localization of ABCG2.

### Effect of Y_6_ on the Intracellular Accumulation of [^3^H]-Mitoxantrone

**FIGURE 5:**
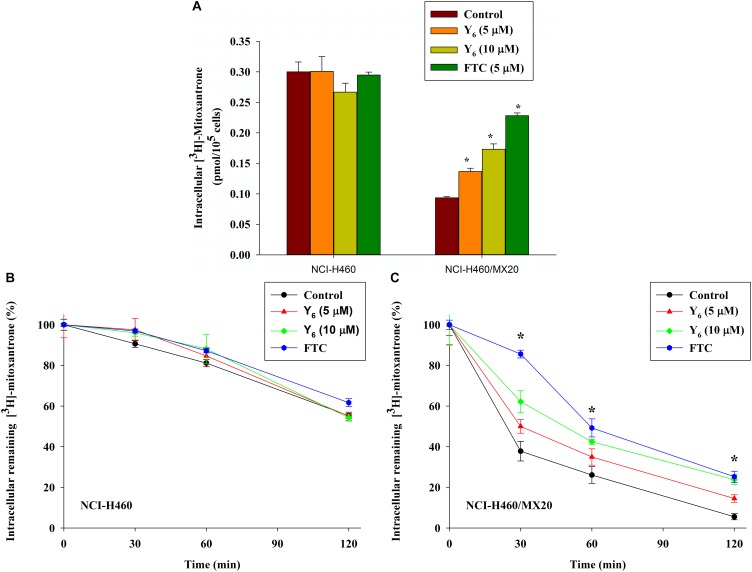
**(A)** Effect of Y_6_ on the accumulation of [^3^H]-mitoxantrone in NCI-H460 and NCI-H460/MX20 cells. ^∗^*p* < 0.05 vs. the control group. FTC at 5 μM is used as positive control for ABCG2-overexpressing cells. **(B,C)** Effect of Y_6_ on the efflux of [^3^H]-mitoxantrone in different times. After 0, 30, 60, or 120 min, the same numbers of NCI-H460 **(B)** and NCI-H460/MX20 **(C)** were placed in the scintillation fluid to measure the radioactivity. Data points represent the means ± SD of triplicate determinations. Experiments were performed at least three independent times. ^∗^*p* < 0.05 vs. control group. FTC at 5 μM was used as a positive control for ABCG2-overexpressing cells.

To further investigate the mechanism of reversal effect, we studied the intracellular accumulation of [^3^H]-mitoxantrone in NCI-H460/MX20 cells treated by Y_6_. We found that the intracellular accumulation of [^3^H]-mitoxantrone was significantly increased in NCI-H460/MX20 cells treated with 5.0 and 10 μM of Y_6_ (Figure 5A). The accumulation increased with increasing concentrations of Y_6_. However, Y_6_ did not show a significant effect on the intracellular accumulation of [^3^H]-mitoxantrone in parental NCI-H460 cells. The results showed that Y_6_ has comparable effects to FTC (5 μM). It could be concluded that Y_6_ significantly increased intracellular concentrations of chemotherapeutic drugs in NCI-H460/MX20 cells and increased cytotoxicity in ABCG2-overexpressing cells.

### Effect of Y_6_ on the [^3^H]-Mitoxantrone Efflux Time-Course

The ABCG2 transporter is known to induce antitumor drug resistance by pumping out drugs and lowering their intracellular concentration. To further measure if the drug accumulation was relative with Y_6_ inhibiting ABCG2-mediated drug efflux, we performed a time-course efflux of [^3^H]-mitoxantrone with or without Y_6_ at different time points. After removing [^3^H]-mitoxantrone from the culture medium, efflux occurred in NCI-H460 and NCI-H460/MX20 cells as shown in Figures 5B,C. After NCI-H460/MX20 cells were incubated for 30 min, about 50, 38, and 62% normalized loss of [^3^H]-mitoxantrone occurred in Y_6_-treated (5, 10 μM) groups and the control group, respectively. About 65, 58, and 73% normalized loss of [^3^H]-mitoxantrone, respectively, occurred with or without Y_6_ after incubating for 60 min; furthermore, NCI-H460/MX20 cells lost about 86, 76, and 94% normalized [^3^H]-mitoxantrone, respectively, after incubating for 120 min. Meanwhile, 10 μM of Y_6_ retained more [^3^H]-mitoxantrone in NCI-H460/MX20 cells than 5 μM of Y_6_ (Figure 5C). Efflux pattern of NCI-H460 cells were not significantly altered by Y_6_ (Figure 5B). The results showed that Y_6_ inhibited the efflux of [^3^H]-mitoxantrone in NCI-H460/MX20 cells and the retention amount in cells followed a Y_6_-concentration-dependent pattern.

### Effect of Y_6_ on the ABCG2 ATP Hydrolysis

**FIGURE 6:**
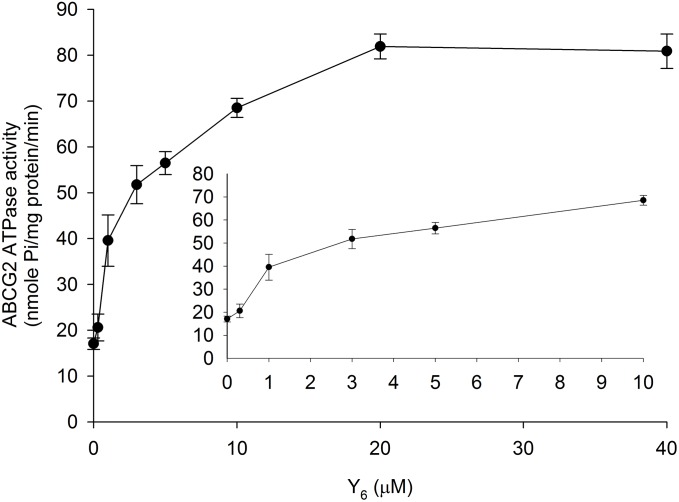
Effect of Y_6_ on the ABCG2 ATPase activity. The Vi-sensitive ATPase activities of ABCG2 in membrane vesicles were determined with different concentrations of Y_6_ (0–40 μM). Experiments were repeated three times.

The ABCG2 transporter could transport substrates across the membrane by utilizing energy derived from ATP hydrolysis. To evaluate the effect of Y_6_ on the ABCG2 ATP hydrolysis, we measured ATPase activity in the presence of Y_6_ at a series of concentrations ranging from 0 to 40 μM. Results showed that Y_6_ stimulated the ATPase activity of ABCG2 at concentrations ranging from 0 to 20 μM. No further increase in ATPase activity occurred when the concentration of Y_6_ was above 20 μM (Figure 6), which could indicate that Y_6_ stimulates the ATPase activity of ABCG2 by acting on the drug-substrate-binding site.

### Docking Analysis of Y_6_ With Human ABCG2 Model

**FIGURE 7:**
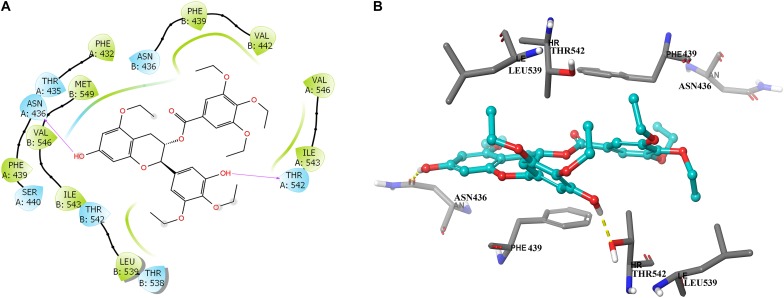
Predicted binding positions of compound Y_6_ with the human ABCG2 transporter protein. **(A)** A two-dimensional ligand–receptor interaction diagram shows the important interactions of compound Y_6_ with the binding site residues of human ABCG2. The amino acids are shown as colored bubbles, cyan indicates polar residues and green indicates hydrophobic residues. Hydrogen bonds are indicated by the purple arrow. **(B)** Docked position of compound Y_6_ within the binding site of the human ABCG2 transporter protein. Compound Y_6_ is shown as a ball and stick model, with the atoms colored as follows: carbon = cyan, hydrogen = white, and oxygen = red. The carbons of the important residues are indicated by the gray color while the other atoms are depicted as sticks with the same color scheme as above and nitrogen is indicated by the blue color. Hydrogen bonds are indicated by dotted yellow lines.

To further learn if Y_6_ have direct interactions with the ABCG2 transporter, we performed a molecular docking simulation. A two-dimensional ligand-receptor interaction diagram of the best-scored induced-fit docked position of Y6 within the drug-binding cavity of human ABCG2 is shown in Figure 7A, while the three-dimensional ligand-receptor interaction diagram is shown in Figure 7B. The 7-hydroxy group in the chroman ring of compound Y_6_ forms a hydrogen bonding with Asn436 (-OH…O=C-Asn436), while the hydroxy group in the 3,4-diethoxy-5-hydroxyphenyl group forms a hydrogen bonding with Thr542 (-OH…OH-Thr542). Moreover, compounds Y_6_ was stabilized into a hydrophilic pocket formed by residues Phe432, Phe439, Val442, Leu539, Ile543, Val546, and Met549 of the ABCG2 protein. From the results, compound Y_6_ had a strong interaction (-11.448 kcal/mol) with the human ABCG2 transporter protein.

## Discussion

In recent years, the studied reversal agents which acted on ABC transporter family mostly targeted ABCB1, and very few targeted ABCG2. FTC was one of the ABCG2 reversal agents that was widely used in ABCG2-mediated MDR research. FTC was a toxin obtained from *Aspergillus fumigatus*, which could inhibit ABCG2 activity at a low concentration without much effect on the functions of ABCB1 and ABCC1. However, its use was limited in clinical patients because of its central nervous system neurotoxicity, although FTC was a classic ABCG2 reversal agent in experiments (Rabindran et al., 1998). Therefore, to look for more safe and effective ABCG2 reversal agents, we performed research on Y_6_ and evaluated its reversal effect on ABCG2-overexpressing cells. Our previously published study indicated that Y_6_ could reverse ABCB1-mediated multidrug resistance by down-regulating ABCB1 protein expression level and changing its function in ABCB1-overexpressing cells (Wen et al., 2017). In this study, we performed the experiments on ABCG2-overexpressing cells and found that Y_6_ was also effective in inhibiting ABCG2-mediated drug resistance at non-toxic concentrations.

In our experiments, mitoxantrone-selected MDR cells, NCI-H460/MX20, and their parental cells, as well as ABCG2-overexpressing transfected cells, HEK293/ABCG2-482-R2, and their parental cells were used to determine cytotoxicity and reversal effect of Y_6_. Results showed that Y_6_ significantly sensitized NCI-H460/MX20 and HEK293/ABCG2-482-R2 to mitoxantrone, SN-38, or topotecan (Tables 1, 2). However, Y_6_ did not increase the cytotoxic effect of cisplatin, which is not a substrate for the ABCG2 transporter. The results implied that Y_6_ could reverse ABCG2-mediated drug resistance in ABCG2-overexpressed cells.

ABCG2 played an important role in protecting tumor cells from cytotoxic damage of antineoplastic drugs (Polgar et al., 2008). One of the most prominent MDR mechanisms is ABCG2 overexpression. The overexpression of ABCG2 can produce MDR to antineoplastic drugs as shown in many studies (Sargent et al., 2001; Ho et al., 2008). Susceptibility to drugs could be increased with absence of ABCG2 as shown in the bcrp1 (-/-) knockout mice studies (Kanzaki et al., 2001; Jonker et al., 2002). Increased ABCG2 expression was also correlated with complete remission, overall survival, relapse-free survival and disease-free survival (Suvannasankha et al., 2004; Damiani et al., 2006; van den Heuvel-Eibrink et al., 2007). To find out the possible mechanism of Y_6_ in reversing ABCG2-mediated drug resistance, we measured its effect on the expression of ABCG2. In our previous study, Y_6_ could down-regulate the expression of ABCB1 transporter in the reverse resistance experiment (Wen et al., 2017). In this study, the expression of ABCG2 transporter was not changed by Y_6_ with different concentrations and incubated time (Figure 3). Y_6_ also did not alter the subcellular localization of ABCG2 in NCI-H460/MX20 cells with different incubated times compared to untreated control (Figure 4). The results indicated that the MDR reversal mechanism of Y_6_ was not relative with the expression level and subcellular localization of ABCG2.

A decreased concentration of intracellular chemotherapeutic drug was a major cause of MDR in cancer cells. In order to further understand the reversal mechanism of Y_6_, we conducted a radiolabeled [^3^H]-mitoxantrone accumulation and efflux study to evaluate the intracellular drug level. ABCG2 is characterized as a part of self-defense systems and function as an efflux pump to transfer the toxic endogenous molecules and xenobiotics, including many cancer chemotherapies, out of the cell (Nakanishi and Ross, 2012). Mitoxantrone is one of ABCG2 substrates as confirmed in studies (Ozvegy et al., 2001; Nakanishi et al., 2003). Cellular resistance to mitoxantrone conferred by ABCG2 could lead to increased efflux and a low level intracellular accumulation of mitoxantrone (Doyle et al., 1998; Miyake et al., 1999; Nakanishi et al., 2003; Robey et al., 2003). Our results showed that NCI-H460/MX20 cells had lower level intracellular accumulation of [^3^H]-mitoxantrone than parental NCI-H460 cells. Y_6_ could increase the accumulation of [^3^H]-mitoxantrone compared to untreated control in the NCI-H460/MX20 cells, and the accumulation followed a Y_6_-concentration-dependent pattern (Figure 5A). Subsequently, an efflux assay was performed to determine whether the accumulation increase was relative to the inhibition of ABCG2 function by Y_6_. The results showed that ABCG2-mediated [^3^H]-mitoxantrone efflux was suppressed by Y_6_ and the efflux amount showed a negative correlation with the concentration of Y_6_ (Figures 5B,C). ABCG2 transfer function was inhibited by Y_6_.

To further understand the interaction of Y_6_ with ABCG2, an ATPase assay using ABCG2 overexpressed membranes and molecular docking simulation using human ABCG2 model were performed. ABC transporters could use energy from the hydrolysis of ATP to transport their substrates across the membrane (Borths et al., 2002; Locher, 2004; Locher and Borths, 2004). It was observed in the study that Y_6_ could stimulate ATPase ability of ABCG2 in a dose-dependent pattern at concentrations ranging from 0 to 20 μM, and then leveled off when Y_6_ concentration was above 20 μM (Figure 6). Moreover, the docking analysis of Y_6_ to human ABCG2 model further revealed that Y_6_ had a strong direct interaction with ABCG2 (Figure 7). Thus, we can confer that Y_6_ stimulates the ATPase ability of ABCG2 by acting on the drug-substrate-binding site.

## Conclusion

The study demonstrated that Y_6_ could reverse ABCG2-mediated MDR *in vitro*. The mechanisms of Y_6_ in reversing MDR is related with the inhibition efflux function of ABCG2 by being a typical competitive substrate of ABCG2, while not relative to down-regulating the ABCG2 expression or altering subcellular localization of ABCG2. Thus, Y_6_ is expected to be a potential ABCG2-mediated resistance reversal agent with multiple targets.

## Author Contributions

Y-AX, Z-SC, and GL contributed to experiment design. R-QZ, YW, PG, Z-NL, and C-YC performed the experiments. R-QZ and YW analyzed the data and wrote the initial draft of manuscript. D-HY reviewed the manuscript. Z-SC and YW obtained the funding.

## Conflict of Interest Statement

The authors declare that the research was conducted in the absence of any commercial or financial relationships that could be construed as a potential conflict of interest.
